# Immunoregulatory Cells in Myasthenia Gravis

**DOI:** 10.3389/fneur.2020.593431

**Published:** 2020-12-15

**Authors:** Ying Wu, Jie Luo, Oliver A. Garden

**Affiliations:** Department of Clinical Sciences and Advanced Medicine, School of Veterinary Medicine, University of Pennsylvania, Philadelphia, PA, United States

**Keywords:** myasthenia gravis, regulatory T cells (Treg), follicular, circulating, regulatory B cells (Breg)

## Abstract

Myasthenia gravis (MG) is a T cell-dependent, B-cell mediated autoimmune disease caused by antibodies against the nicotinic acetylcholine receptor or other components of the post-synaptic muscle endplate at the neuromuscular junction. These specific antibodies serve as excellent biomarkers for diagnosis, but do not adequately substitute for clinical evaluations to predict disease severity or treatment response. Several immunoregulatory cell populations are implicated in the pathogenesis of MG. The immunophenotype of these populations has been well-characterized in human peripheral blood. CD4^+^FoxP3^+^ regulatory T cells (Tregs) are functionally defective in MG, but there is a lack of consensus on whether they show numerical perturbations. Myeloid-derived suppressor cells (MDSCs) have also been explored in the context of MG. Adoptive transfer of CD4^+^FoxP3^+^ Tregs or MDSCs suppresses ongoing experimental autoimmune MG (EAMG), a rodent model of MG, suggesting a protective role of both populations in this disease. An imbalance between follicular Tregs and follicular T helper cells is found in untreated MG patients, correlating with disease manifestations. There is an inverse correlation between the frequency of circulating IL-10–producing B cells and clinical status in MG patients. Taken together, both functional and numerical defects in various populations of immunoregulatory cells in EAMG and human MG have been demonstrated, but how they relate to pathogenesis and whether these cells can serve as biomarkers of disease activity in humans deserve further exploration.

## Introduction

Myasthenia gravis (MG) is a chronic autoimmune disease characterized by muscle weakness and fatigue (1, 2). Pathogenic autoantibodies in MG target components of the post-synaptic muscle endplate located at the neuromuscular junction, impairing neuromuscular transmission (3). A vast majority of patients have antibodies against muscle nicotinic acetylcholine receptors (AChRs); a minority have antibodies against muscle-specific tyrosine kinase (MuSK) or low-density lipoprotein receptor-related protein 4 (LRP4) (2, 4). MG patients without detectable autoantibodies are referred to as having seronegative MG. Apart from autoantibody specificity, MG can be subclassified based on age of onset, clinical presentation, and thymic histopathology (3, 5). Heterogeneity of the disease makes predicting prognosis challenging (1, 6). Conventional treatment options, including symptomatic treatments and general immunosuppression, can help many but not all patients (5). Durable remission remains improbable, and chronic treatment with high doses of non-specific immunosuppressive drugs is usually necessary to maintain disease remission. Current therapeutic approaches lack specificity and are associated with a number of side effects (1, 5). Identifying new biomarkers that can predict disease progression and treatment response and can be practically applied in clinical studies is highly desirable for the development of more specific and better tolerated treatments for MG patients.

The primary outcome measure of choice in MG trials is so far focused on the effect of clinical signs and symptoms (7). Single fiber electromyography represents the most robust biomarker of neuromuscular transmission, but is limited by factors related to accuracy, reproducibility, and availability of technical expertise (8). Antibody titers to AChR or MuSK have been used as a marker of the therapeutic response, but the correlation of this measure with disease severity has not been confirmed (9–12). Attempts to identify new biomarkers face challenges. Serum metabolomic profiling distinguishes patients with anti-AChR antibody-seropositive (AChR+) MG from those without (13), but whether metabolic analysis can predict therapeutic outcome remains to be explored.

Immunoregulatory cells operate in the periphery to modulate immune responses, especially those of autoreactive T and B cells that have escaped central tolerance (14–17). They are implicated in the pathogenesis of a variety of autoimmune diseases, including MG (18–21). Regulatory cells can be readily phenotyped and isolated on the basis of surface antigens and have been reported in a number of studies of MG (22–36). This review summarizes current knowledge of regulatory cells in MG, including their potential implication in pathogenesis.

## Immunoregulatory Cells

Immunoregulatory cell populations are diverse in their lineage and phenotype. Regulatory cells in the lymphoid lineage are represented by regulatory T (Tregs) (37–39), regulatory B (Bregs) (20, 40), and regulatory natural killer cells (41–43), while those in the myeloid lineage comprise myeloid-derived suppressor cells (MDSCs) (44–47), regulatory dendritic cells (DCs) (48–50), regulatory macrophages (51–53), regulatory neutrophils (54–57), and regulatory eosinophils (58, 59).

### Regulatory T Cells

#### CD4^+^FoxP3^+^ Regulatory T Cells

**Figure 1:**
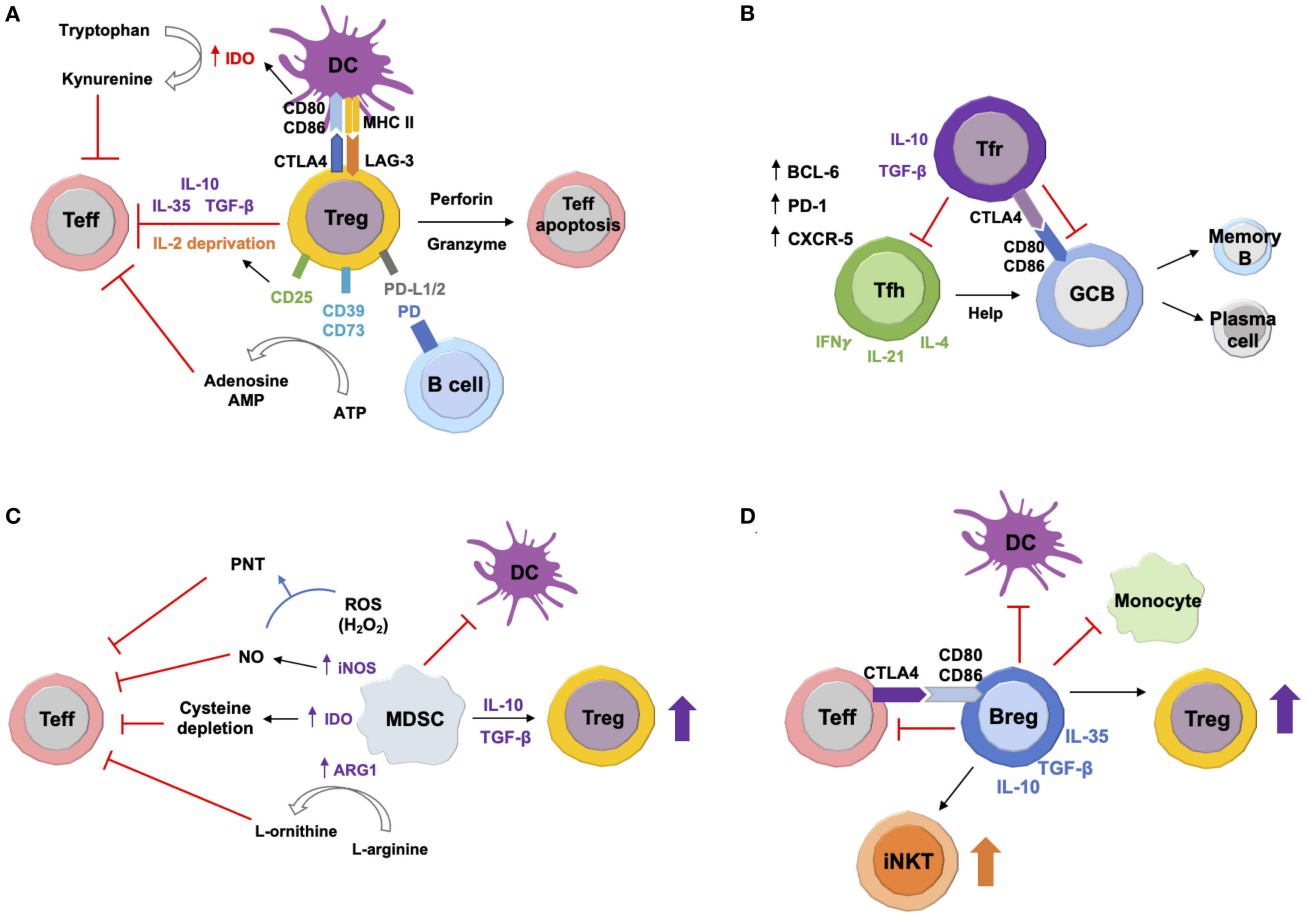
Suppressive Mechanisms of Immunoregulatory Cells. **(A)** Tregs secrete anti-inflammatory cytokines IL-10, transforming growth factor beta (TGF-β), and IL-35, thus inhibiting target effector T (Teff) cells. Production of perforin and granzyme causes Teff cell apoptosis. Constitutively high expression of CD25 depletes IL-2, which attenuates Teff cell proliferation. Interaction with dendritic cells (DCs) *via* cytotoxic T-lymphocyte-associated protein 4 (CTLA-4) and lymphocyte-activation gene 3 (LAG-3) downregulates CD80/CD86 expression, which induces upregulation of indoleamine 2,3-dioxygenase (IDO). This enzyme expressed by DCs converts tryptophan to kynurenine, leading to Teff cell exhaustion. Surface expression of CD39 and CD73 converts extracellular adenosine triphosphate (ATP) to immunosuppressive adenosine and adenosine monophosphate (AMP). Tregs can also suppress autoreactive B cells *via* programmed death (PD) ligands 1 and 2 (PD-L1/2). **(B)** In germinal centers (GCs), both follicular helper T (Tfh) and follicular regulatory T (Treg) cells express transcription factor B cell lymphoma 6 (BCL6), surface marker PD-1, and C-X-C motif chemokine receptor 5 (CXCR5). Tfh cells produce IL-4, IL-21, and interferon gamma (IFNγ). They provide help signals to GC B cells and promote their differentiation into antibody-secreting plasma cells and memory B cells. Tfr cells regulate GC responses by inhibiting both Tfh and B cells *via* anti-inflammatory IL-10 and TGF-β. Tfr cells can also directly suppress GC B cells *via* CTLA-4. **(C)** Myeloid-derived suppressor cells (MDSCs) produce high levels of inducible nitric oxide synthase (iNOS), arginase-1 (ARG1), and reactive oxygen species (ROS). iNOS generates nitric oxide (NO), which reacts with ROS to produce peroxynitrite (PNT). ARG1 converts L-arginine to L-ornithine. IDO expressed by MDSCs sequesters cysteine. All of these can inhibit Teff cells. MDSCs also induce Treg expansion *via* IL-10 and TGF-β. In addition, MDSCs suppress maturation, migration, and antigen presentation of DCs. **(D)** Regulatory B cells (Bregs) inhibit activation and differentiation of pro-inflammatory target cells, including Teff cells, DCs and monocytes *via* secretion of IL-10, IL-35, and TGF-β. Bregs can also directly suppress Teff cells *via* CTLA-4 and CD80/CD86 interaction. On the other hand, Bregs induce expansion and differentiation of Tregs and invariant natural killer T (iNKT) cells. (Suppressive mechanisms in this figure refer to general contexts, including homeostasis and all inflammatory conditions.)

As a principal player in peripheral tolerance, Tregs are among the most widely studied of the regulatory cells (38, 39). In humans and mice, Tregs are characterized as suppressive T cells, predominantly CD4^+^, that constitutively express CD25 and the transcription factor forkhead box P3 (FoxP3) (37, 60). Human CD4^+^CD25^+^FOXP3^+^ T cells are heterogeneous and have been labeled by additional surface antigens such as CD127, CD45RA/RO, and sialyl lewis x (CD15s) to further delineate naïve Tregs as CD25^+^CD127^low^CD45RA^+^FoxP3^low^, activated Tregs as CD25^high^CD127^low^CD45RA^−^FoxP3^high^, highly suppressive Tregs as CD25^+^CD127^low^CD45RA^−^CD15s^+^FoxP3^+^, and non-suppressive T cells (also known as “non-Tregs”) as CD25^+^CD127^low^CD45RA^−^FoxP3^low^ (61– 66). Tregs have also been identified in domestic animal species, including dogs and cats (67–72), which are gaining traction as spontaneous models for many human diseases (73–79). Our previous work has revealed a conserved transcriptomic signature of Tregs among humans, mice, and dogs, vindicating the view that these cells are phenotypically and functionally related between these mammalian taxa. Thirty-one consensus transcripts were highly expressed in Tregs of all three species in comparison with their conventional T cell counterparts. Of the 31 consensus transcripts, six encode the Treg signature molecules CD25, FoxP3, IL-10, Helios, Galectin 3, and T-cell immunoreceptor with immunoglobulin and immunoreceptor tyrosine-based inhibitory motif domains (TIGIT) (68). Many other T cell subsets possess regulatory function, including CD8^+^ T cells (80–82), type 1 regulatory T (Tr1) cells (83, 84), γδ T cells (85, 86), and invariant natural killer T (iNKT) cells (87, 88). However, CD4^+^FoxP3^+^ Tregs (Figure 1A and Table 1) dominate research in this field (17, 21, 89).

**Table 1:** Summary of Immunoregulatory Cells in AChR+ MG.

**Cell types**	Markers^*^	Cytokines^*^	Target cells^*^	**Association with pathogenesis in MG**	**References**
CD4^+^FoxP3^+^ Treg	CD25^+^ or CD25^high^, FoxP3, CTLA-4 (CD152), GITR, LAG-3, Neuropilin-1, CD127^−/low^, Sialyl Lewis x (CD15s)	IL-10, TGF-β, IL-35	Teff cells, APCs, B cells	- Functional defect is associated with reduced FoxP3 expression and MG pathogenesis; - Decreased FoxP3 expression correlates with attenuated STAT5 signaling; - Numerical correlation remains controversial; - Adoptive transfer treats EAMG	(22–35, 37, 61, 64, 130)
Tfh	CD4^+^CXCR5^+^PD-1^+^/ICOS^+^	IL-21, IL-4, IL-17, IFNγ	GC B cells	- Cell frequency positively correlates with disease severity; - Tfr/Tfh ratio inversely correlates with disease severity	(102–113)
Tfr	CD4^+^CXCR5^+^FoxP3^+^	IL-10, TGF-β	Tfh cells; GC B cells	- Cell frequency inversely correlates with disease severity; - Tfr/Tfh ratio inversely correlates with disease severity	(98–101, 107, 112, 113, 131)
PMN-MDSC	CD11b^+^CD14^−^CD15^+^CD33^+^ or CD11b^+^CD14^−^CD66^+^CD33^+^ (human); CD11b^+^Ly6G^+^Ly6C^low^ (mouse); CD11b^+^CD14^−^CADO48^+^ (dog)	IL-10, TGF-β	Teff cells; DCs; macrophages	Adoptive transfer of MDSC treats EAMG in mice	(44–47, 115, 123)
M-MDSC	CD11b^+^CD14^+^CD15^−^CD33^+^HLA-DR^−/low^ (human); CD11b^+^Ly6G^−^Ly6C^high^ (mouse); CD11b^+^CD14^+^CADO48^−^ (dog)	IL-10, TGF-β	Teff cells; DCs; macrophages	Adoptive transfer of MDSC treats EAMG in mice	(44–47, 115, 123)
Breg	CD19, CD38, CD1d, CD24, CD27	IL-10, TGF-β	Teff cells; DCs; monocytes; iNKTs	Cell frequency and function inversely correlate with disease severity	(20, 36, 40, 124, 125)

* *Markers, cytokines, and target cells refer to general contexts, including homeostasis and all inflammatory conditions*.

*FoxP3, forkhead box P3; CTLA-4, cytotoxic T-lymphocyte-associated protein 4; GITR, glucocorticoid-induced tumor necrosis factor receptor; LAG-3, lymphocyte-activation gene 3; TGF-β, transforming growth factor beta; Teff, effector T cells; APCs, antigen-presenting cells; STAT5, signal transducer and activator of transcription 5; CXCR5, C-X-C motif chemokine receptor 5; PD-1, programmed death 1; ICOS, inducible T cell co-stimulator; IFNγ, interferon gamma; GC, germinal center; DCs, dendritic cells; iNKTs, invariant natural killer T cells*.

A number of studies have characterized Tregs in human AChR+ MG patients based on CD25 and/or FoxP3 expression (22, 23, 25–32, 35). A majority of reports found no alteration in the frequency or absolute number of Tregs isolated from either peripheral blood or thymus of MG patients in comparison with those from healthy subjects (22, 23, 25, 26, 28, 29, 31, 32, 35). However, some studies made disparate observations. In the study of Fattorossi et al. (30), the number of circulating Tregs in untreated MG patients was lower than those in healthy subjects and MG patients treated with prednisone and azathioprine, which suggested that the clinical benefit of immunosuppressive therapy may in part be attributable to increasing Treg numbers. These authors also found that although thymectomy transiently inhibited the increase in frequency of circulating Tregs following immunotherapy, circulating Tregs in these patients eventually returned to a level similar to those of patients treated with immunotherapy without thymectomy. These data suggest that circulating Treg recovery during immunotherapy might be independent of the thymus. Li et al. (27) found lower frequency of circulating CD4^+^ Tregs, but unaltered frequency of CD8^+^ Tregs, in MG patients than in healthy controls. However, further studies with subgroup analysis is needed to discern the difference between the subtypes of MG and the effect of medications. In contrast to the lack of consensus on numerical perturbations of Tregs in MG, impaired function of Tregs has been consistently demonstrated by *in vitro* functional analysis (22, 23, 26, 28, 29, 32, 35). The dysfunction has been associated with attenuated FoxP3 expression, given the pivotal role of FoxP3 in Treg development and function (90–92). One study suggested a link between decreased FoxP3 expression and lowered phosphorylation of signal transducer and activator of transcription-5 (STAT5) (35). Furthermore, Luther et al. (26) reported that Tregs from prednisolone-treated MG patients had enhanced suppressive function *in vitro* compared to those from untreated patients, suggesting that prednisolone might augment Treg function. This result accords with the findings of Fattorossi et al. (30), which also showed augmentation of Treg numbers during immunosuppressive medication. Together, these data indicate a potential role of immunosuppressive therapy in restoring Treg number and function. However, both studies only compared treated and untreated patients at a single time point. A longitudinal study is needed to address this hypothesis. In addition, stability of Treg function is likely to be influenced by the inflammatory environment in MG. For instance, the inflammatory cytokine tumor necrosis factor alpha (TNF-α) negatively modulates human CD4^+^CD25^high^ Treg function (93). A more recent study showed that loss of FoxP3 expression by human Tregs mediated by TNF-α depends on the FoxP3 complex component Deleted in Breast Cancer 1 (DBC1) (94).

Studies on experimental autoimmune MG (EAMG) in rodents have provided additional insight into the role of Tregs. Aricha et al. (34) showed that myasthenic rats had a lower frequency of circulating CD4^+^CD25^high^FoxP3^+^ T cells than healthy controls, while Nessi et al. (24) found no difference in frequency of CD4^+^CD25^+/high^ T cells in either spleens or lymph nodes between rats with EAMG and healthy controls. Both groups also investigated the therapeutic effect of passive transfer of Tregs. Aricha et al. (33, 34) reported that adoptive transfer of *in vitro*-induced polyclonal Tregs from either healthy or EAMG donors suppressed ongoing EAMG. Nessi et al. (24) found that CD4^+^CD25^+^ T cells isolated from naïve rats prevented the induction of EAMG, but did not suppress established disease. This observation might reflect insufficient numbers of activated Tregs among administered CD4^+^CD25^+^ T cells.

Only a limited number of studies have investigated T cell populations in peripheral blood of human patients with MuSK+ MG. Yi et al. (95) found that CD4^+^ T cells exhibit enhanced inflammatory Th1 and Th17 responses in MuSK+ MG, although no difference was found in either frequencies or CD39 expression of FoxP3^+^ Tregs between MuSK+ MG and healthy controls, suggesting that increased pro-inflammatory T cell responses were not attributed to numerical or functional defects of Tregs. The same group (96) also reported that tacrolimus, an immunosuppressant for AChR+ MG, inhibited Th1 and Th17 responses, and reduced Treg frequencies of *in vitro* cultured peripheral blood mononuclear cells (PBMCs) from MuSK+ MG patients. Reuveni et al. (97) reported that a mouse model of MuSK+ EAMG had decreased Treg frequencies and FoxP3 expression, the latter of which was restored by oral administration of recombinant MuSK protein.

In summary, AChR+ MG is associated with functional defects of Tregs. Adoptive transfer of Tregs derived from either healthy rats or myasthenic rats can attenuate EAMG. In contrast, the pathogenic role of Tregs remains unclear in MuSK+ MG.

#### Follicular Regulatory T Cells

Follicular regulatory T (Tfr) cells (Figure 1B and Table 1) have emerged as a unique subset of CD4^+^ Tregs that negatively regulate the proliferation of follicular helper T (Tfh) and B cells in germinal centers (GCs) (98, 99). Both Tfr and Tfh cells express common GC-associated antigens, including transcription factor B cell lymphoma 6 (BCL6), chemokine receptor CXCR5, programmed death-1 (PD-1), and inducible T-cell co-stimulator (ICOS) (100–103). However, unlike Tfh cells, Tfr cells concurrently express Treg-characteristic markers such as CD25, FoxP3, glucocorticoid-induced tumor necrosis factor receptor (GITR), and cytotoxic T-lymphocyte antigen 4 (CTLA-4) (100, 101). Tfr and Tfh cells regulate humoral immunity in opposite directions (104). Imbalance between these two populations dysregulates production of autoantibodies, promoting pathogenic autoimmunity (105, 106). Tfr and Tfh cells primarily reside in GCs (98). However, some studies have identified counterpart CD4^+^ T cell subsets in peripheral blood, facilitating investigation of their pathogenic potential in the context of autoimmunity, including MG (107, 108).

The frequency of a population of CD4^+^CXCR5^+^ T cells was higher in the peripheral blood of untreated MG patients than in that of healthy controls (109). The cell frequency was positively correlated with disease severity. Thymectomy followed by glucocorticoid therapy reduced CD4^+^CXCR5^+^ T cell frequency in these myasthenic patients to the control level (109). In a similar observation, an increased frequency of circulating Tfh cells, defined as CD4^+^CXCR5^+^PD-1^high^/ICOS^high^ cells, was demonstrated in MG patients in comparison to healthy subjects (110). The study also showed a positive correlation between circulating Tfh cell frequency and serum anti-AChR antibody titer in these patients (110). In line with these clinical studies, EAMG mice also have a higher frequency of splenic CD4^+^CXCR5^+^PD-1^+^ Tfh cells than controls, and their Tfh cell frequency is positively correlated with the concentration of anti-AChR antibodies in serum (111). All these findings collectively suggest that the frequency of circulating Tfh cells reflects disease activity in AChR+ MG. However, a shortcoming in these studies is the lack of distinction of Tfr and Tfh cells amongst circulating follicular T cells.

Three recent studies showed that AChR+ MG patients had a lower frequency of circulating Tfr cells, but a higher frequency of circulating Tfh cells than healthy controls, suggesting a link between the imbalance of the Tfr/Tfh ratio and disease manifestations (107, 112, 113). The Tfr/Tfh ratio showed an inverse correlation with AChR+ MG severity, and the imbalance was restored by steroid and cyclophosphamide therapy (107). Taken together, the ratio between circulating Tfr and Tfh cells is likely to predict the development of AChR+ MG. Similarly, a higher Tfh/Tfr ratio was found in MuSK+ MG patients, accompanying increased frequencies of Th17-producing Tfh cells and higher Tfh-promoted IgG synthesis (114). The pathological roles of Tfr and Tfh populations in MG need to be further investigated in animal models.

### Other Regulatory Cell Populations

In contrast to Tregs, limited information is available on other regulatory populations in MG. To date, only MDSCs and Bregs have been examined in MG.

#### Myeloid-Derived Suppressor Cells

MDSCs (Figure 1C and Table 1) are a heterogeneous population of immature myeloid cells that accumulate in cancers and other diseases involving chronic inflammation (45). These cells suppress T cell responses and contribute to tumor progression and metastasis, emerging as a promising therapeutic target in cancer (46). MDSCs comprise two major subsets, polymorphonuclear (PMN)- and monocytic (M)-MDSCs (47). They are distinguished from conventional neutrophils or monocytes by surface antigens and density (44, 47). In humans, PMN-MDSCs are identified as CD11b^+^CD14^−^CD15^+^CD33^+^ or CD11b^+^CD14^−^CD66b^+^CD33^+^ hypodense myeloid cells, while M-MDSCs are identified as CD11b^+^CD14^+^CD15^−^CD33^+^HLA-DR^−/low^ hypodense myeloid cells; both populations are found in the PBMC fraction after density gradient separation (44). The murine counterparts of PMN- and M-MDSCs are CD11b^+^Ly6G^+^Ly6C^low^ and CD11b^+^Ly6G^−^Ly6C^high^ cells, respectively (44). Our previous work has identified functional equivalents of these subsets in dogs based on the expression of CADO48A and CD14 (115). The role of MDSCs has been investigated in a variety of autoimmune diseases (116–122). The ability of *in vitro* generated MDSCs to suppress EAMG has been investigated in mice (123). Adoptive transfer of MDSCs improved muscle weakness, reducing both serum anti-AChR IgG levels and complement deposition at the endplates in EAMG mice. Splenocytes from MDSC-treated mice had a lower production of IFN-γ and IL-17 *in vitro*, demonstrating reduced Th1 and Th17 responses. MDSCs also directly inhibited pre-activated B cells both *in vitro* and *in vivo*. These results suggest that MDSCs suppress ongoing EAMG by inhibiting both autoreactive T and B cells (123).

#### Regulatory B Cells

Bregs (Figure 1D and Table 1) have been identified in humans and mice as a heterogeneous population of immunosuppressive B cells that inhibit pro-inflammatory responses predominantly by means of IL-10 synthesis (40, 124). However, intracellular staining for IL-10 precludes functional studies of Bregs, prompting Breg isolation using surface markers such as CD19, CD38, CD24, CD1d, and CD27 (20, 40). Breg frequency and function are negatively correlated with disease activity of several autoimmune disorders, such as systemic lupus erythematosus (SLE), rheumatoid arthritis (RA), and multiple sclerosis (MS) (20). Two studies have shown reduced frequency and function of circulating Bregs in untreated AChR+ MG patients compared with healthy controls (36, 125). The proportion of circulating Bregs can be restored by thymectomy, but not by steroid therapy (125). A subset of Bregs, namely IL-10–producing B (B10) cells (126), repopulated at a faster rate in the patients with a favorable response to rituximab than in those with a poor response (36). In addition, Guptill et al. (127) also reported a reduction of B10 frequencies in MuSK+ MG patients compared to healthy controls. These results together suggest an immunopathogenic role of diminished Bregs in both AChR+ and MuSK+ MG. Adoptive transfer of Bregs has not yet been reported in MG. However, Bregs transferred into mice with experimental autoimmune encephalomyelitis induced FoxP3^+^ Tregs and Tr1 cells, and correlated with disease remission (128). This observation suggests that Bregs might hold promise as an adoptive cellular therapy for MG.

## Discussion

Current data suggest that immunoregulatory cells may play significant roles in the pathogenesis of MG. In AChR+ MG patients, these populations show either functional defects (CD4^+^FoxP3^+^ Tregs) or numerical deficiency (Tfr), or both (Bregs). They can be readily isolated from patients' peripheral blood and characterized by flow cytometry. Performing functional assays in the current routine clinical setting can be challenging, while numerical analysis of circulating Tfr, Tfh, or Breg cells shows promising utility in clinical practice. However, several drawbacks need to be addressed before these assays may be translated for clinical use.

First, current studies have extensively examined AChR+ MG cases, leaving a scarcity of knowledge for the less common, but equally debilitating, MuSK+, LRP4+, and seronegative phenotypes of MG — although the nature of a small subpopulation of a rare disease makes such studies challenging. Second, the current studies have treated all AChR+ MG patients as a homogeneous group, calling into question whether these assays can further differentiate subsets of MG patient groups, including classification based on clinical presentation, age of onset, gender, and thymic histopathology. Third, the low frequencies of circulating Tfr and Breg cells are a significant obstacle in accurate quantification of these populations. An alternative is to analyze the characteristic gene expression of these populations by qRT-PCR assay. Furthermore, antigen-specific regulatory cells may closely correlate with disease severity in MG, assessed using MHC-peptide tetramers or fluorescently-labeled antigens (129).

In conclusion, numerical measures of circulating Tfr, Tfh and B10 cells appear to correlate with disease activity of AChR+ MG; however, none of these populations shows sufficient sensitivity or specificity to serve as a biomarker for the disease. Mechanistic insight into the roles of immunoregulatory cells in the pathogenesis of MG will enable the development of more targeted therapies for this debilitating autoimmune disease in the future.

## Author Contributions

YW conceptualized and drafted the manuscript. OG and JL critically reviewed the manuscript. All authors contributed to the article and approved the final version.

## Conflict of Interest

The authors declare that the research was conducted in the absence of any commercial or financial relationships that could be construed as a potential conflict of interest.
